# Surgical margins after partial nephrectomy as prognostic factor for the risk of local recurrence in pT1 RCC: a systematic review and narrative synthesis

**DOI:** 10.1007/s00345-022-04016-0

**Published:** 2022-05-03

**Authors:** Michaël M. E. L. Henderickx, Suraj V. Baldew, Lorenzo Marconi, Marcel D. van Dijk, Faridi S. van Etten-Jamaludin, Brunolf W. Lagerveld, Axel Bex, Patricia J. Zondervan

**Affiliations:** 1 Department of Urology, Amsterdam UMC location University of Amsterdam, Meibergdreef 9, Amsterdam, The Netherlands; 2 Department of Urology, Coimbra University Hospital, Coimbra, Portugal; 3 Faculty of Medicine (AMC), University of Amsterdam, Amsterdam, The Netherlands; 4 Research Support, Medical Library, Amsterdam UMC location University of Amsterdam, Meibergdreef 9, Amsterdam, The Netherlands; 5 Department of Urology, OLVG, Amsterdam, The Netherlands; 6 The Royal Free London NHS Foundation Trust and UCL Division of Surgery and Interventional Science, London, UK; 7 Department of Urology, The Netherlands Cancer Institute, Antoni Van Leeuwenhoek Hospital, Amsterdam, The Netherlands

**Keywords:** Local recurrence, pT1 renal cell carcinoma, Partial nephrectomy, Prognostic factor, Systematic review, Positive surgical margin

## Abstract

**Purpose:**

To systematically review the published literature on surgical margins as a risk factor for local recurrence (LR) in patients undergoing partial nephrectomy (PN) for pT1 renal cell carcinomas (RCC).

**Evidence acquisition:**

A systematic literature search of relevant databases (MEDLINE, Embase and the Cochrane Library) was performed according to the PRISMA criteria up to February 2022. The hypothesis was developed using the PPO method (Patients = patients with pT1 RCC undergoing PN, Prognostic factor = positive surgical margins (PSM) detected on final pathology versus negative surgical margins (NSM) and Outcome = LR diagnosed on follow-up imaging). The primary outcome was the rate of PSM and LR. The risk of bias was assessed by the QUIPS tool.

**Evidence synthesis:**

After assessing 1525 abstracts and 409 full-text articles, eight studies met the inclusion criteria. The percentage of PSM ranged between 0 and 34.3%. In these patients with PSM, LR varied between 0 and 9.1%, whereas only 0–1.5% of LR were found in the NSM-group. The calculated odds ratio (95% confident intervals) varied between 0.04 [0.00–0.79] and 0.27 [0.01–4.76] and was statistically significant in two studies (0.14 [0.02–0.80] and 0.04 [0.00–0.79]). The quality analysis of the included studies resulted in an overall intermediate to high risk of bias and the level of evidence was overall very low. A meta-analysis was considered unsuitable due to the high heterogeneity between the included studies.

**Conclusion:**

PSM after PN in patients with pT1 RCC is associated with a higher risk of LR. However, the evidence has significant limitations and caution should be taken with the interpretation of this data.

**Supplementary Information:**

The online version contains supplementary material available at 10.1007/s00345-022-04016-0.

## Introduction

According to the AUA and EAU guidelines, partial nephrectomy (PN) is currently the standard of care in cT1a and most cT1b RCC [1, 2]. The major drawback of PN is the risk of positive surgical margins (PSM). Until today, there is an on-going debate concerning the clinical relevance of PSM on local recurrence (LR). Therefore, patients with PSM might be included in a comprehensive follow-up schedule, including extensive imaging regimens for early detection of any (local) recurrences. Patients in this group are also considered to be more likely to need secondary local therapies [3]. Although little is known about the optimal treatment modality for PSM (radical nephrectomy or focal therapy) and the timing of treatment [4].

The influence of surgical margin on LR in T2 RCC and higher has been investigated in the past, and findings suggest an increased risk of LR in patient with PSM after PN [5–7]. LR was found in 16% of patients with PSM after PN and only occurred in three percent of patients with negative surgical margins (NSM). Even more, two systematic reviews, one by Minervini et al*.* (Minerva Urol. E Nephr.; 2017) and one more recent by Hakam et al*.* (Urology.; 2021), investigated the effect of PSM on the oncological outcome [8, 9]. However, both systematic reviews included all T-stages. To the best of our knowledge, no systematic reviews have been published that solely included pT1-stage RCC’s. Thus, leaving studies investigating the prognostic value of surgical margins on LR in pT1 RCC after PN remain scarce and it is still unclear if PSM result in a higher risk of LR.

This study aims to systematically review the literature on surgical margins as risk factors for LR in patients undergoing PN for pT1 RCC and provide a complete overview of the current evidence on this subject.

Evidence acquisition:

### Data acquisition and search strategy

This review was conducted in accordance with the PRISMA statement [10]. The review protocol was registered and published on the international prospective register of systematic reviews (PROSPERO) with registration number CRD42020162793. The PRISMA checklist can be found in Supplementary Appendix 1.

The hypothesis was developed using the PPO-method (Patients, Prognostic factor, and Outcome) and was defined as follows:

- Patients with pT1 RCC submitted to PN (*Patients*),
- Prognostic factor: PSM (detected on final pathology) versus NSM,
- Outcome: LR diagnosed on follow-up imaging.

A systematic literature search of studies published up to February 2020 was performed by our institutions medical librarian, covering three electronic databases: MEDLINE (PubMed), Embase (Ovid) and the Cochrane library. An update of this search was performed in February 2022, to screen data that were published during the process of writing this review. The search strategy can be found in Supplementary Appendix 2. Only studies published in the English language were screened. Case reports, letters to the editor, editorials, congress abstracts and studies in paediatric patients were excluded. The literature search was completed by searching the reference lists of the included studies for potentially relevant studies and additional reports identified by the author panel.

All abstracts and full-text articles were independently reviewed by two reviewers (M.M.E.L.H. and S.V.B.), following the pre-defined inclusion and exclusion criteria to retrieve relevant articles. Any disagreement regarding the inclusion of an article was resolved based on consensus. If consensus could not be reached, a third and fourth independent reviewer (L.M. and P.J.Z.) were asked to make a final judgment.

### Types of studies and participants

Retrospective and prospective cohort studies describing surgical margins and LR in patients with pT1 RCC who underwent PN, were eligible for inclusion. However, studies including pT2 RCC and higher and not differentiating between pT1 and ≥ pT2 RCC in their outcomes, were excluded. Furthermore, only histologically proven pT1 RCC were included for analysis. And finally, patients who received salvage radical surgery after the detection of PSM were also excluded.

If several papers were reporting on the same outcome using identical study cohorts, the data from the most recent paper was used. However, if different outcomes from identical study cohorts were reported, data from all relevant publications were collected and analyzed independently.

### Prognostic factors evaluated

The surgical margin of the PN specimen as assessed by a pathologist on final pathology (as a categorical variable) was evaluated. PMSs were defined as tumor in contact with the sample’s stained area in histological evaluation.

### Types of outcome measures included

The primary outcome of this review was LR (as an absolute number of events). It was defined as tumor bed recurrence or recurrence near the site of the original tumor in the ipsilateral kidney. Diagnosis of LR was based on radiological imaging.

### Measures of association

Primary outcome and measure of association were extracted from the included studies. Relative differences were used as the measure of the relationship between surgical margin and LR. These differences were based on the odds ratios. Associations were calculated to be in the same direction, with estimates > 1 indicating a poorer prognosis relative to the best prognosis group.

### Assessment of risk of bias and level of evidence

Included papers were critically appraised to assess the overall risk of bias by two investigators (M.M.E.L.H. and P.J.Z.) using the Quality in Prognosis Studies (QUIPS) tool for non-randomized studies [11]. Six categories were evaluated: participants, attrition, prognostic factor measurement, outcome measurement, study confounding, and statistical analysis, and reporting. Disagreement was resolved based on consensus. If consensus could not be reached, a third reviewer (S.V.B.) was asked to make a final judgment.

The GRADE (Grading of Recommendations, Assessment, Development and Evaluations)-tool for grading the quality of evidence was used to assess the level of evidence of the included studies [12]. Five categories were evaluated: risk of bias, imprecision, inconsistency, indirectness, and publication bias to determine an overall level of evidence. An overall GRADE quality rating for the level of evidence was determined by taking the lowest quality of evidence from all of the outcomes [13].

### Data synthesis

Data were extracted and recorded in Microsoft^®^ Excel for Mac V.2016 (Microsoft Corp, Redmond, WA, USA) by a single investigator (M.M.E.L.H.) and subsequently checked by three other investigators (S.V.B., L.M. and P.J.Z.). Baseline study characteristics were extracted and presented using descriptive statistics. Initially, this review was intended as a meta-analysis if valid data assessing the association between surgical margin and LR were available from sufficiently homogeneous studies concerning population, prognostic factor, outcome and definitions. However, because of great heterogeneity between the different studies, no meta-analysis, but a narrative synthesis was performed.

Review Manager (RevMan) Version 5.3. (Copenhagen: The Nordic Cochrane Centre, The Cochrane Collaboration) was used to create the simple Forest plot.

## Evidence synthesis

### Quantity of evidence synthesis

**Fig. 1:**
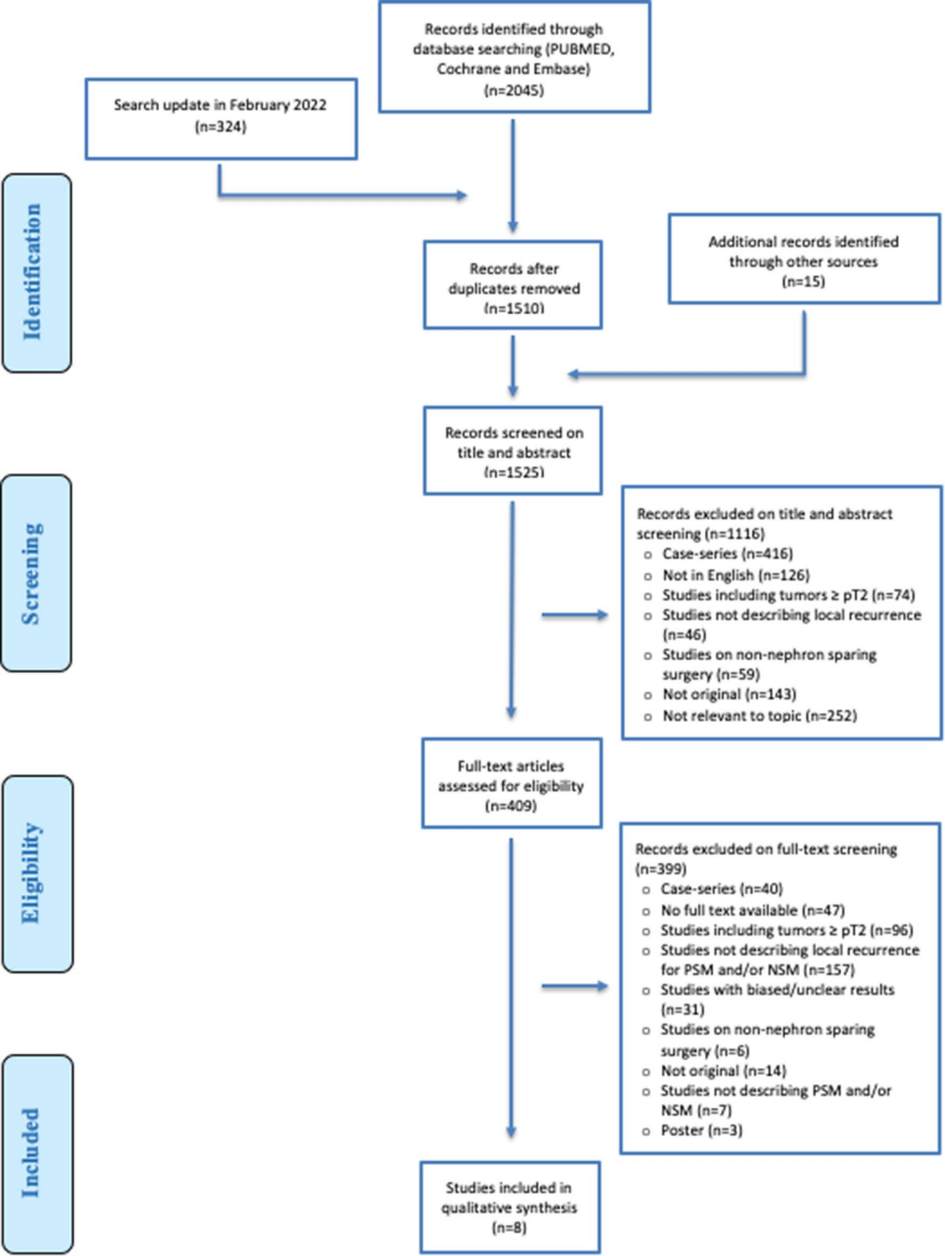
PRISMA flowchart

The literature search identified a total 2045 papers. The search update performed in February 2022, identified an additional 324 papers. In these 2369 papers, 859 duplicates were detected and removed and 15 additional records were identified through other sources, resulting in 1525 papers left for screening. The title and abstract of these 1525 papers were screened for eligibility. Consequently, a total of 1116 articles were excluded. The remaining 409 papers were assessed for eligibility on full-text screening. This resulted in the selection of eight eligible studies [14–21]. The screening process of the studies is summarized according to the PRISMA flow chart (Fig. 1).

The baseline characteristics of the included studies are shown in Table 1. Table 2 presents the definitions used to define PSM, LR, as well as the used follow-up scheme. The included studies were published between 2016 [14] and 2021 [21]. A total of 4040 procedures were described in the eight included papers. The median follow-up ranged from 24 months (IQR 12–40 months) to 61 months (range 48–76 months) [17, 18]. Radfar et al*.*, who only found LR in the PSM-group, described a mean time to LR of 9 months ± 6.3 months (range 2 to 18 months) [21]. The median age of the included patients varied between 52 to 62 years. Finally, seven studies reported on the presence of metastasis in their study cohort [15–21].

**Table 1:** Characteristics of the included studies

#	Author	Study type	Type of procedure	Tumor size (cm)	Follow-up (m)	Procedures	% PSM	% LR in PSM	% LR in NSM
1	Kang et al*.* [14]	RC	NR	2.8 ± 1.1/2.5 ± 1.1 (mean ± SD)	32.5 (median)	1813	1.7%	0%	0.4%
2	Oh et al*.* [15]	RC	OPN and RAPN	2.3 + 0.8/2.2 + 0.8 (mean + SD)	48.3 (median)	702	1.6%	0%	0.3%
3	Li et al*.* [16]	RC	OPN and LPN	*NR*	56 (median)	600	3.3%	0%	0.2%
4	Marchiñena et al*.* [17]	RC	OPN, LPN and RAPN	2.9 (2.1–3.8) (median (IQR))	24 (12–40) median (IQR))	314	7%	9.1%	1.4%
5	Minervini et al*.* [18]	RC	RAPN	3.0 (2.0–3.7) (median (IQR))	61 (48–76) (median (range))	121	2.5%	0%	0%
6	Çinar et al*.* [19]	RC	OPN and LPN	3.53 ± 1.29/3.01 ± 1.08 (mean + SD)	28.9 (mean)	215	7.6%	6.3%	1.5%
7	Wu et al*.* [20]	RCT	LPN	3.0 (1.0–4.0)/3.0 (1.5–4.0) (median (range))	24 (median)	15	0%	0%	0%
8	Radfar et al*.* [21]	RC	OPN and LPN	*NR*	32.3 ± 28.0/32.1 ± 25.9 (mean + SD)	122	34.4%	11.9%	0%

*RC* retrospective cohort, *RCT* randomized controlled trial, *RAPN* robot-assisted partial nephrectomy, *OPN* open partial nephrectomy, *LPN* laparoscopic partial nephrectomy, *m* months, *IQR* interquartile range, *SD* standard deviation, *PSM* positive surgical margin, *LR* local recurrence, *NSM* negative surgical margin

**Table 2:** Definitions used for PSM, LR and follow-up in the included studies

#	Author	Definiton of PSM	Definition of LR	Definition of follow-up
1	Kang et al*.* [14]	No clear definition	Tumor recurrence at the site of the previous PN	History, physical examination, routine blood work and serum chemistry, chest X-ray and CT abdomen every 3–6 months. Elective bone scan, MRI or PET-CT on indication
2	Oh et al*.* [15]	Malignant cells being present at the inked parenchymal surgical margin of resection on the final pathology assessment	Tumor bed recurrence	CT abdomen, blood test and chest x-ray at 3 months, 12 months and annually thereafter
3	Li et al*.* [16]	Large number of residual tumor cells at the surgical margin OR incision of satellite tumor nodules around the large tumor	In situ recurrence	The follow-up was specific to each institution’s practice with a physical examination and a CT scan of the abdomen usually included
4	Marchiñena et al*.* [17]	Tumor cell that contacted with Chinese ink	Tumor mass in the ipsilateral kidney over the resection bed of the same histological type of the original tumor	The oncologic follow-up was performed according to the NCCN guidelines
5	Minervini et al*.* [18]	Presence of neoplastic cells directly in contact with the inked surface of the specimen	Recurrence at the enucleation site was considered true LR	Physical examination, routine laboratory tests and CT chest and abdomen at 6 months and then yearly for the first 5 years
6	Çinar et al*.* [19]	The presence of malignant cells at the surgical margin	No clear definition	Complete physical examinations and serum creatinine measurements were done at the postoperative first, third and twelfth months and annually thereafter. Plain chest X-rays and Ct scans of the abdomen were obtained in the sixth postoperative month and annually after that
7	Wu et al*.* [20]	No clear definition	No clear definition	Follow-up was conducted at 3 and 6 months after surgery and every 6 months thereafter. It involved chest and abdomen CT scanning or MRI imaging
8	Radfar et al*.* [21]	Extension of the tumor to the surface of the specimen in permanent pathology	New detection of the tumor mass in the same surgery site based o radiographic evidences on chest X-ray, CT scan, MRI or bone scan with or without pathologic confirmation	History, physical examination, blood tests, chest X-ray and abdominal-pelvic CT scan every 6 to 12 months in the first 5 years and then annually

*PSM* positive surgical margin, *LR* local recurrence

### Analysis of surgical margins as a prognostic factor for local recurrence

Kang et al*.* described the largest study cohort of 1813 pathology-proven clear cell RCC. They did not find a significant difference in LR between PSM and NSM (*p* = 0.492). Furthermore, they did not find a statistically significant difference in tumor grade between the PSM and NSM-group in their study (*p* = 0.141). Moreover, Kang et al*.* postulated that SM was not associated with recurrence-free survival on Kaplan–Meier analysis (*p* = 0.566) [14].

Oh et al*.* studied the surgical margin-width after open and robot-assisted PN. They found that the surgical margins in patients with recurrence was smaller (2.26 ± 1.51 mm), when compared with patients without recurrence (2.43 ± 2.07 mm; *p* = 0.218). However, they did not specifically investigate the difference in LR between PSM and NSM. They also described tumor grade for their entire study cohort, but did not correlate this with surgical margin after PN or LR [15].

Li et al*.*, investigated 600 patients undergoing PN in China to create a classification for PSM. They distinguished between false positive SM and true PSM. The authors performed a Fisher’s exact test resulting in a significant higher recurrence rate between PSM and NSM (*p* = 0.0252), as well as true PSM and NSM (p = 0.0094), but not between false PSM and NSM (*p* = 0.3727). They only described tumor grade for the patients with PSM in their study cohort, but did not correlate these findings with LR [16].

Marchiñena et al*.* described that the LR-free survival three years post-PN was significantly higher (*p* = 0.02) in the negative SM-group (96.4% (95% CI 91.9–100)) when compared to the PSM-group (87.8% (95% CI 71.9–100)). Furthermore, they also performed a multivariate analysis and found that PSM (hazard ratio 12.9, 95% CI 1.8–94, *p* = 0.011) and Fuhrman grade III or IV (hazard ratio 38.3, 95%CI 3.1–467, p = 0.004) were independent predictors of LR [17].

The study of Minverini et al*.* was the smallest series included and looked at oncologic outcomes after robot-assisted tumor enucleation. They focused on the predictors for pseudocapsule invasion and distinguished between absent, partial and complete pseudocapsule invasion. Interestingly, all PSM had completely invaded pseudocapsules. Their study described tumor grade for their entire study cohort, but did not correlate this with surgical margin after PN or LR [18].

In their study, Çinar et al*.* described their experience with laparoscopic PN for T1a RCC. They compared a cohort of patients that underwent open PN with a cohort that underwent laparoscopic PN and found no statistical significant difference in PSM-rate between both groups. Furthermore, they described four patients with LR (one with PSM in the laparoscopic group, two with NSM in the laparoscopic group and one with NSM in the open group). They explicitly described the characteristics of their four patients with LR. Surprisingly, the patients with a high grade tumor and LR all had NSM (*n* = 3), whereas the patient with a low grade tumor and LR had a PSM after PN (*n* = 1) [19].

Wu et al*.* was the only of the included studies that performed a randomized controlled trial. They compared laparoscopic microwave assisted enucleation with laparoscopic PN. They had 21 patients (13.8%) with high grade tumors in their study cohort. All patients in this study had NSM and no LR or metastasis were found within a median follow-up of 24 months [20].

Finally, Radfar et al*.*, looked at the data of 750 patients that underwent PN at their center between 2004 and 2018. They included 42 patients with pathology-proven RCC and PSMs after PN and matched these patients with 80 patients with NSM after PN. They found LR in 5 patients from the PSM-group, while no LR was found in the NSM-group. Thus, in their study, LR occurred more in the PSM-group. Nonetheless, this did not affect the overall survival when compared with the NSM-group. Remarkable, all recurrences and metastasis belonged to the subtype of clear cell RCC. Furthermore, they did not find a statistically significant difference in tumor grade between the PSM and NSM-group (*p* = 0.601). They also found that there was no significant difference in tumor grade between the patients with and without LR (*p* = 0.612). However, they did not specifically look into the association of PSM in patients with high grade tumors and their possible influence on LR [21].

Surgical margins were described in all eight studies as dichotomous variables [14–21]. PSMs were assessed by a pathologist on final pathology and malignant cells present in contact or present in the (inked) parenchymal surgical margin were defined as PSM. Detailed information on how a PSM was defined can be found in Table 2. Only Kang et al*.* and Wu et al*.* did not provide a clear definition for a PSM [14, 20]. The percentage of PSM ranged between 0 and 34.4% in the included studies. In these patients with PSMs, LR varied between 0–9.1%, whereas only 0–1.5% of LR were found in the NSM-group.

**Fig. 2:**
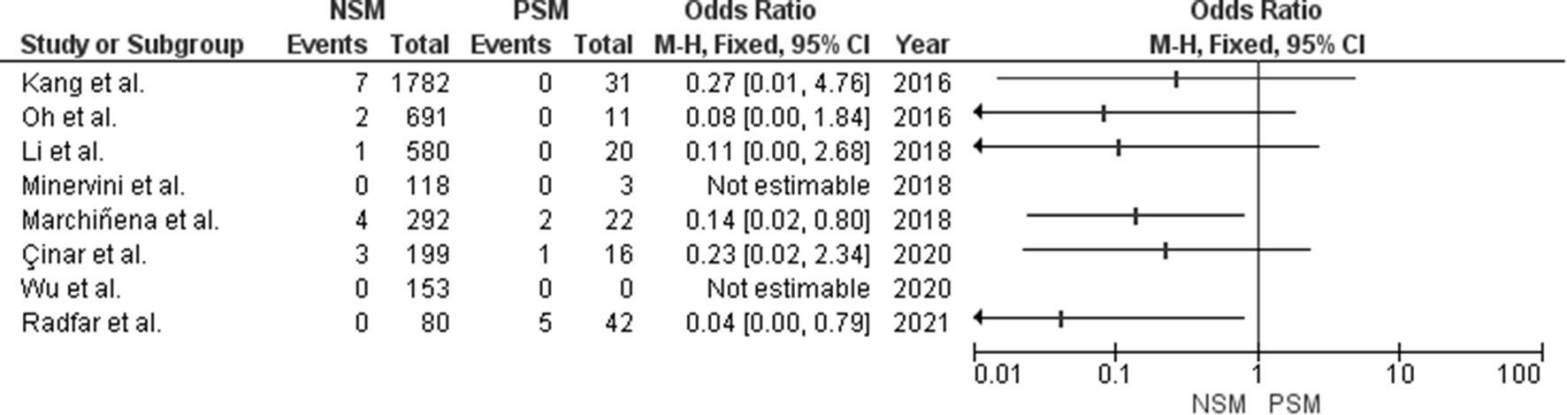
Forest plot

All studies had complete data on surgical margin and LR-rate available, which made it possible to calculate the odds ratios and to compute a simple Forest plot (Fig. 2). Six studies provided raw numbers on surgical margins in patients with and without LR, enabling us to calculate the corresponding odds ratio and construct a simple Forest plot [14–17, 19, 21]. However, the odds ratio was only statistically significant in two studies [17, 21]. These two significant results indicate a decreased occurrence of LR or a protective exposure for the presence of NSM.

### Risk of bias and quality assessment

Relevant information describing population, patient selection and methodology was frequently lacking, resulting in a moderate to high risk of bias across the six domains. Therefore, overall studies were considered to have a high risk of bias (Table 3).

**Table 3:**
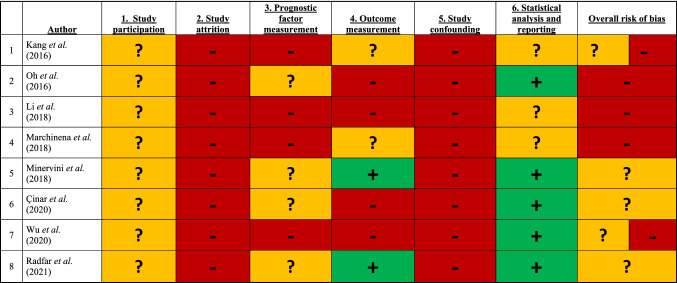
Risk of bias using QUIPS tool (Green = low risk; Yellow = unknown/intermediate risk; Red = high risk)

### Level of evidence assessment

Because all but one of the included studies were retrospective cohort studies, there was consequent residual confounding. Therefore, the level of evidence of these studies, that included observational data, started at a low quality level. Due to the high risk of bias of the included studies, certainty in evidence was rated down to very low (Table 4).

**Table 4:**
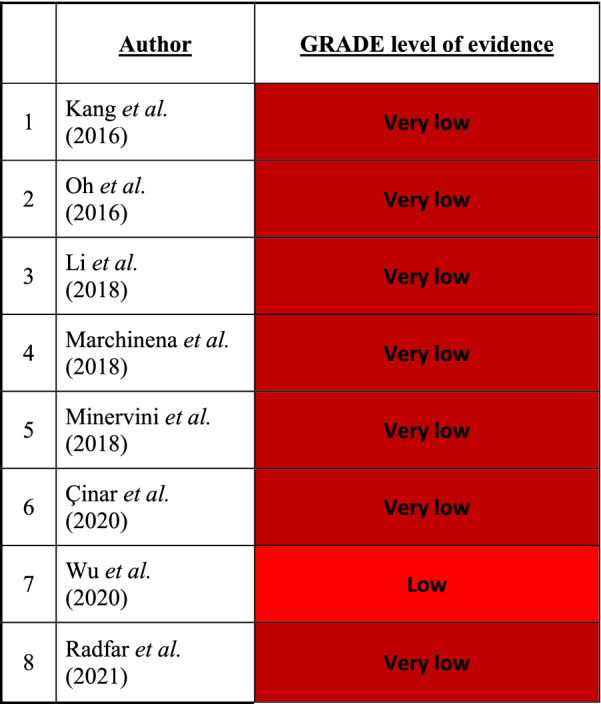
Level of evidence based on the GRADE) tool for the included studies

## Discussion

We hypothesized that a PSM may have a significant higher rate of LR after PN for pT1 RCC.

This systematic review of the literature found important outcomes. A total of eight studies was included and the percentage of PSM ranged between 0 and 34.4% in the included studies. In these patients with PSM, LR varied between 0–9.1%, whereas only 0–1.5% of LR were found in the NSM-group. Odds ratio was statistically significant in two of the included studies and indicated a protective exposure for the presence of NSM.

The findings of this systematic review are comparable to what was found in previous studies. Malik Wahba et al*.* found that PSM substantially increases LR (3/12 patients vs. 8/362 patients) in patients with PSM after robot-assisted PN, however, overall survival was not affected (*p* = 0.13) in their study with 77 months of follow-up [22]. A systematic review by Minervini et al., assessing the effect of surgical technique (simple enucleation versus standard partial nephrectomy) on surgical margins, whereas this systematic review focused on the effect of PSM as a risk factor for LR, irrespective of the technique used. More important, Minervini et al*.* allowed studies investigating all T-stages in their inclusion criteria, whereas this review focused exclusively on pT1-tumors [8]. The same is true for a recent study by Hakam et al*.* who performed a systematic review on the effect of PSM nephron sparing surgery on oncological outcome in for all RCC’s [9]. Hakam et al*.* found an increased risk of LR (hazard ratio of 6.11 – high certainty), whereas Minervini et al*.* described a LR rate of less than 5% after PN [8, 9]. The latter is comparable to the findings of this systematic review, which focused exclusively on the subgroup of pT1 RCC’s.

The analysis in this review were based on cases that underwent PN only, so no confounders will be found regarding technical restraints. However, there may be a difference between the various types of PN. In this regard, the possibility of a significant discrepancy between for instance, open PN and laparoscopic PN, cannot be ruled out. Nonetheless, studies comparing laparoscopic PN and open PN, found no difference in progression free survival, overall survival, margin status and LR-rate [23, 24]. Furthermore, most studies did not describe a nephrometry-score (PADUA or RENAL) for the included cases to assess the anatomical complexity of the treated tumor. A higher complexity may affect surgical outcome and thus confound with our results. Nonetheless, mean or median tumor size was emphatically described in six of the eight included papers (Table 1) and pT-stage was described in all studies. Nevertheless, a univariate and multivariate analysis performed by Khalifey et al*.* could not identify any factor (tumor size, growth pattern, pathological stage, tumor grade, multiple tumors or surgeon learning curve) that significantly predicted PSM [25]. Different studies postulate that high tumor grade is correlated with worse oncologic outcomes in RCCs [26–28]. Kwon et al*.* for example, described that tumors with high malignant potential and PSM have a higher risk for LR [29]. Minervini et al*.* looked at the difference in cancer-specific survival between low and high tumor grade and found that tumor grade is important for predicting long-term survival. However, this statement was especially true for ≥ pT2 tumors [30]. Sorbellini et al*.* searched for prognostic factors to predict recurrence after surgery for clear cell RCC. They found that tumor grade and lymphovascular invasion was an independent predictor of recurrence and developed a nomogram to predict the 5-year recurrence-free survival [31]. More recently, Takagi et al*.* included non-clear cell RCCs, when they looked for predictive factors for recurrence after PN for cT1a RCCs. They investigated patients' characteristics and tumor factors of 1227 patients and found that high grade tumors and upstaging to pT3a are two independent factors that predict worse recurrence-free survival [32].

Schiavina and colleagues then again, investigated which variables were predictors for PSM after PN and also developed a nomogram. In their study, surgical approach was an independent predictor for PSM. This statement is backed by the findings of Minervini et al*.*, who found that resection techniques significantly impacted the risk for PSM after PN [33]. All but one of the included studies, described the surgical approach, but resection technique was only described in two of the included studies [18, 19]. Kang et al. even stated that the surgical technique varied widely in their study. Knowing that the surgical approach and resection technique can potentially influence the surgical margin status and PSM then again, might predict a worse recurrence-free survival; this could lead to spurious results and might complicate the interpretation.

Another very interesting, although still controversial topic, is the approach for PSM as well as the management of LR [21]. All included papers chose to perform active surveillance for their patients with PSM, which is in line with studies who have shown that the presence of PSM did not affect overall survival [21, 34]. Bensalah and colleagues looked into the percentage of true PSM and found that residual tumor was only found in 39% of the patients who underwent auxiliary surgery. Radical nephrectomy or re-resection of PSM can therefore result in over-treatment in many cases. Consequently, the EAU guidelines recommend to counsel patients with PSM about the increased risks and state that they need a more intense follow-up [2].

As for the management of LR, unfortunately, none of the included papers described their management for the patients with LR. The EAU guidelines recommend to offer local treatment of locally recurrent disease when it is technically possible and significant comorbidities are absent, however this recommendation has a weak strength rating [2].

Brassetti et al*.* introduced a novel trifecta for robot-assisted PN based on standardized parameters to improve reliability in reproducibility. In this study, the authors performed a retrospective analysis of a multicenter, multi-national dataset of patients with non-metastatic cT1-2 RCC and found that this newly defined trifecta (NSM, no major (Clavien-Dindo ≥ 3) complications and ≤ 30% postoperative eGFR reduction) represents a significant predictor for recurrence, mortality and deterioration of the renal function [35].

Our comprehensive systematic review investigated the influence of surgical margin on LR focused exclusively on pT1 RCC and showed a difference in LR between the PSM-group and the NSM-group, favoring the latter. One of the strengths is the systematic approach to analyze the prognostic value of surgical margin as risk factor for the development of LR in patients with pT1 RCC. The methodology included Cochrane reporting standards, such as PRISMA and the standardized “QUIPS” critical appraisal tool for non-randomized studies tool to assess risks of bias and the GRADE tool to grade the quality of evidence of the included studies.

Limitations, however, include the reported results of the included articles. All but one of the included papers were retrospective cohort studies and are therefore subject to the bias that is inherent to these kinds of studies. Furthermore, this systematic review was not based on papers containing the highest level of evidence available for medical research [36]. However, cohort studies are the only ethically sound method to assess the natural history of patients with PSM, therefore, the available data analyzed in this systematic review and narrative synthesis are of the highest achievable level of evidence on this subject [37]. Consequentially, risk of bias in the included papers is high. Furthermore, the heterogeneity between the included studies is high. This is especially true for the used definition of PSM, LR as well as for the follow-up scheme as shown in Table 2. Not only did the definitions differ, but also the interpreter of the results differed between studies (one dedicated pathologist or radiologist versus different pathologists and radiologists). These different approaches could significantly influence both the validity and the reproducibility of the study results.

Another limitation is the median follow-up in the included studies. The median follow-up of the included studies ranged from 24 months (IQR 12–40 months) to 61 months (range 48–76 months). Although most LRs occur in the first two years after surgery (Radfar et al*.* for example describes a mean time to LR of 9 months (range 2–18 months), LR can also occur much later [21]. As shown by Tellini et al*.*, who found a median time to LR for PSM of 43 months (IQR 17–68 months) and for NSM of 56 months (IQR 26–96 months) [38]. A study by Bernhard et al*.* found that LR occurred at a median time of 27 months (IQR 14.5–38.2 months), but states that it can happen much later (up to 209 months after surgery in their study) [39]. Remarkably, Yoo et al*.* showed that papillary RCCs have a higher risk of developing recurrence after at least five years post-surgery, when compared to clear cell RCCs (4.8% versus 0.3%, *p* < 0.001) [40].

Further research on the effect of LR on cancer-specific survival and overall survival is needed to understand the clinical relevance of these findings. This could be a cohort of cases matched to controls by propensity score for analysis of surgical margin after PN for pT1 RCC specifically, with a long follow-up to assess the effect of LR on overall and cancer-specific survival. Interestingly, a study that includes the specific location of the margin (parenchymal versus perinephric), grade at the margin, and positive margin length would be of interest to further specify these factors of relevance. Possible prognosticators may be found to help deciding whether a PSM should be treated by salvage therapy or not [7, 41].

## Conclusion

This comprehensive systematic review showed that a PSM is related to a higher rate of LR after PN for pT1 RCC. Nonetheless, the evidence for surgical margins as a prognostic value for LR has significant limitations due to the low level of evidence and high heterogeneity of the included studies. Thus, caution should be taken with the interpretation of these data and further research on this topic is needed.

## Supplementary Information

Below is the link to the electronic supplementary material.

Supplementary file1 (PDF 835 KB)

Supplementary file2 (DOCX 16 KB)

## Data Availability

The template data collection forms, data extracted from included studies, data used for all analyses, analytic code, any other materials used in the review are available on demand with the authors.

## Abbreviations

CI: Confidence interval
IQR: Interquartile range
LR: Local recurrence
PN: Partial nephrectomy
RCC: Renal cell carcinoma
NSM: Negative surgical margin
PSM: Positive surgical margin
