# Supplementary material for: Surgical margins after partial nephrectomy as prognostic factor for the risk of local recurrence in pT1 RCC: a systematic review and narrative synthesis
Source: World J Urol. 2022 May 3;40(9):2169–79. doi: 10.1007/s00345-022-04016-0 (PMC9427912; doi:10.1007/s00345-022-04016-0)
Supplement: Supplementary file 2 — Supplementary file2 (DOCX 16 KB) [file 345_2022_4016_MOESM2_ESM.docx]

**Appendix:**

Appendix 2: literature search

*PubMed:*

("Kidney Neoplasms"[Mesh] OR "Nephrons"[Mesh] OR "Kidney Cortex/pathology"[Mesh] OR nephron*[tiab] OR small renal mass*[tiab] OR T1[tiab] OR cT1a[tiab] OR cT1b[tiab] OR T1a[tiab] OR T1b[tiab] OR renal cell ca*[tiab] OR kidney tumo*[tiab] OR kidney cancer*[tiab] OR kidney mass*[tiab] OR renal tumo*[tiab])

**AND**

("Nephrectomy"[Mesh] OR "Kidney Neoplasms/surgery"[Mesh] OR "Kidney Cortex/surgery"[Mesh] OR "Carcinoma, Renal Cell/surgery"[Mesh] OR "Nephrons/surgery"[Mesh] OR nephrectom*[tiab] OR heminephrectom*[tiab] OR nephron sparing surger*[tiab] OR NSS[tiab])

**AND**

("Margins of excision"[Mesh] OR surgical margin*[tiab] OR margin excision*[tiab] OR excision margin*[tiab] OR resection margin*[tiab] OR positive margin*[tiab] OR incomplete resect*[tiab])

**NOT**

("Letter"[Publication Type] OR "Editorial"[Publication Type] OR "Comment"[Publication Type] OR letter[ti] OR editorial[ti])

*Embase (Ovid):*

Database(s): Embase Classic+Embase 1947 to february 2020

| # | Searches |
| --- | --- |
| 1 | exp kidney tumor/ or exp nephron/ or kidney cortex/ or (nephron* or small renal mass or T1 or cT1a or cT1b or T1a or T1b or renal cell ca* or kidney tumo* or kidney cancer* or kidney mass* or renal tumo*).ti,ab,kw. |
| 2 | nephrectomy/ or exp partial nephrectomy/ or nephron sparing surgery/ or exp kidney tumor/su or exp kidney cortex/su or renal cell carcinoma/su or exp nephron/su or (nephrectom* or heminephrectom* or nephron sparing surger* or NSS).ti,ab,kw. |
| 3 | surgical margin/ or (surgical margin* or margin excision* or excision margin* or resection margin* or positive margin* or incomplete resect*).ti,ab,kw. |
| 4 | 1 and 2 and 3 |
| 5 | limit 4 to conference abstract status |
| 6 | 4 not 5 |
| 7 | editorial/ or letter/ or (letter or comment or editorial).ti. |
| 8 | 6 not 7 |

*Cochrane Library:*

ID Search

#1 (nephron* or small renal mass or T1 or cT1a or cT1b or T1a or T1b or renal cell ca* or kidney tumo* or kidney cancer* or kidney neoplasm* or kidney mass* or renal tumo*):ti,ab,kw

#2 (nephrectom* or heminephrectom* or nephron sparing surger* or NSS): ti,ab,kw

#3 MeSH descriptor: [Margins of Excision] explode all trees

#4 (surgical margin* or margin excision* or excision margin* or resection margin* or positive margin* or incomplete resect*):ti,ab,kw

#5 #3 or #4

#6 #1 and #2 and #5

UPDATE: February 2020 to 11 february 2022

*PubMed*

198 hits:

("Kidney Neoplasms"[Mesh] OR "Nephrons"[Mesh] OR "Kidney Cortex/pathology"[Mesh] OR nephron*[tiab] OR small renal mass*[tiab] OR T1[tiab] OR cT1a[tiab] OR cT1b[tiab] OR T1a[tiab] OR T1b[tiab] OR renal cell ca*[tiab] OR kidney tumo*[tiab] OR kidney cancer*[tiab] OR kidney mass*[tiab] OR renal tumo*[tiab])

AND

("Nephrectomy"[Mesh] OR "Kidney Neoplasms/surgery"[Mesh] OR "Kidney Cortex/surgery"[Mesh] OR "Carcinoma, Renal Cell/surgery"[Mesh] OR "Nephrons/surgery"[Mesh] OR nephrectom*[tiab] OR heminephrectom*[tiab] OR nephron sparing surger*[tiab] OR NSS[tiab])

AND

("Margins of excision"[Mesh] OR surgical margin*[tiab] OR margin excision*[tiab] OR excision margin*[tiab] OR resection margin*[tiab] OR positive margin*[tiab] OR incomplete resect*[tiab])

NOT

("Letter"[Publication Type] OR "Editorial"[Publication Type] OR "Comment"[Publication Type] OR letter[ti] OR editorial[ti])

AND ("2020/01/28"[Date - Publication] : "2022/02/11"[Date - Publication])

*Embase (Ovid):*

Database(s): **Embase Classic+Embase**1947 to 2022 February 10

| # | Searches |
| --- | --- |
| 1 | exp kidney tumor/ or exp nephron/ or kidney cortex/ or (nephron* or small renal mass or T1 or cT1a or cT1b or T1a or T1b or renal cell ca* or kidney tumo* or kidney cancer* or kidney mass* or renal tumo*).ti,ab,kw. |
| 2 | nephrectomy/ or exp partial nephrectomy/ or nephron sparing surgery/ or exp kidney tumor/su or exp kidney cortex/su or renal cell carcinoma/su or exp nephron/su or (nephrectom* or heminephrectom* or nephron sparing surger* or NSS).ti,ab,kw. |
| 3 | surgical margin/ or (surgical margin* or margin excision* or excision margin* or resection margin* or positive margin* or incomplete resect*).ti,ab,kw. |
| 4 | 1 and 2 and 3 |
| 5 | limit 4 to conference abstract status |
| 6 | 4 not 5 |
| 7 | editorial/ or letter/ or (letter or comment or editorial).ti. |
| 8 | 6 not 7 |
| 9 | limit 8 to yr="2020 -Current" |

*Cochrane Library:*

ID Search

#1 (nephron* or small renal mass or T1 or cT1a or cT1b or T1a or T1b or renal cell ca* or kidney tumo* or kidney cancer* or kidney neoplasm* or kidney mass* or renal tumo*):ti,ab,kw

#2 (nephrectom* or heminephrectom* or nephron sparing surger* or NSS):ti,ab,kw

#3 MeSH descriptor: [Margins of Excision] explode all trees

#4 (surgical margin* or margin excision* or excision margin* or resection margin* or positive margin* or incomplete resect*):ti,ab,kw

#5 #3 or #4

#6 #1 and #2 and #5 with Cochrane Library publication
